# Comparative effects of intermittent theta-burst stimulation and sequential bilateral rTMS on depression and emotion regulation in major depressive disorder: a randomized controlled trial

**DOI:** 10.3389/fnins.2026.1756383

**Published:** 2026-02-13

**Authors:** Li Pu, Yaqin Li, Zhengzhi Deng, Chaoli Deng, Anran Hu, Jiang Wu, Zhihong Chen, Dezhong Yao, Hongmei Yan, Guojian Yan

**Affiliations:** 1 The Clinical Hospital of Chengdu Brain Science Institute, Sichuan Institute for Brain Science and Brain-Inspired Intelligence, China-Cuba Belt and Road Joint Laboratory on Neurotechnology and Brain-Apparatus Communication, School of Life Science and Technology, University of Electronic Science and Technology of China, Chengdu, China; 2 The Fourth People’s Hospital of Chengdu, Chengdu, China

**Keywords:** cognitive reappraisal, emotion regulation, intermittent theta-burst stimulation (iTBS), major depressive disorder, sequential bilateral rTMS

## Abstract

**Background:**

Major depressive disorder (MDD) is associated with dysfunction in prefrontal–limbic circuits, particularly involving deficits in emotion regulation. Non-invasive brain stimulation, including sequential bilateral repetitive transcranial magnetic stimulation (rTMS) and intermittent theta-burst stimulation (iTBS), targets these circuits, but comparative efficacy and mechanistic underpinnings remain unclear.

**Methods:**

In this randomized controlled trial, 114 patients with MDD were recruited, and 106 completed the study. Participants were randomly assigned (1:1) to receive either sequential bilateral rTMS (*n* = 50) or iTBS (*n* = 56) over 2 weeks. Primary outcome was change in Hamilton depression rating scale (HAMD) scores. Secondary outcomes included changes in cognitive reappraisal, expressive suppression, anhedonia (SHAPS), anxiety (HAMA), and other psychobehavioral measures. Bonferroni correction was applied to adjust for multiple comparisons across secondary outcome measures. All change scores were calculated as baseline minus post-treatment values. Associations between changes in cognitive reappraisal and depressive symptoms were assessed using generalized linear models.

**Results:**

Baseline demographic and clinical characteristics were comparable between groups. Following treatment, iTBS elicited significantly greater reductions in depressive symptoms (HAMD: 14.88 ± 5.25 vs. 7.88 ± 5.26; *P* < 0.001) and improvements in cognitive reappraisal (−10.36 ± 6.49 vs. −3.54 ± 4.41; *P* < 0.001, significant after Bonferroni correction) compared with sequential bilateral rTMS. iTBS also produced larger decreases in expressive suppression and anxiety. In the iTBS group, improvements in cognitive reappraisal were significantly associated with reductions in depressive symptoms (*B* = −0.324, *P* = 0.001), whereas no such association was observed in the sequential bilateral rTMS group.

**Conclusion:**

Intermittent theta-burst stimulation targeting the left dorsolateral prefrontal cortex induces more pronounced improvements in depressive symptoms and cognitive reappraisal than sequential bilateral rTMS, likely associated with potentiation of left prefrontal emotion-regulatory circuits. Sequential bilateral rTMS also provides therapeutic benefit, possibly through restoring interhemispheric balance.

## 1 Introduction

Major depressive disorder (MDD) is increasingly conceptualized not merely as a disorder of mood, but as a dysfunction of distributed and plastic prefrontal-limbic circuits that support cognitive and emotional control (McGaugh, 2013). Within this framework, a reproducible feature of the illness is an imbalance in functional engagement between left and right prefrontal systems, reflecting disrupted large-scale network organization rather than isolated regional pathology (Gibson et al., 2022). Evidence from neuroimaging, electrophysiology and structural morphology indicates a pattern often summarized as relative left-prefrontal hypoactivity (and structural compromise) alongside either preserved or relatively increased right-prefrontal responsivity. Importantly, this hemispheric imbalance manifests across modalities (fMRI, EEG, cortical excitability) and is increasingly understood as macroscopic manifestations of underlying network-level dysfunction, particularly involving deficits in top-down control over subcortical affective nodes such as the amygdala and hippocampus (Miller et al., 2015; Liu et al., 2017; Brunoni et al., 2017). Recent network-based models of depression further emphasize dysfunction within prefrontal–limbic circuits, including aberrant connectivity between the dorsolateral prefrontal cortex (DLPFC) and subgenual anterior cingulate cortex (sgACC), as well as disrupted integration across frontoparietal and default mode networks (Bennett, 2011; Taylor and Liberzon, 2007). From this perspective, hemispheric imbalance can be viewed as a manifestation of maladaptive network plasticity, rather than a competing explanatory framework. Framing MDD as a syndrome of disturbed hemispheric plasticity clarifies why therapies that restore network-level plasticity-pharmacological, psychotherapeutic or neuromodulatory-produce clinical benefit: symptom resolution often corresponds to restoration of adaptive, experience-dependent changes in prefrontal circuits rather than correction of a unitary chemical deficit (Berwian et al., 2024).

Non-invasive brain stimulation, and specifically repetitive transcranial magnetic stimulation (rTMS) and theta-burst variants, has been developed to act directly upon these prefrontal control systems. Many established clinical protocols are explicitly grounded in the hemispheric imbalance model, aiming to upregulate hypoactive left prefrontal regions and/or downregulate putatively hyperactive right prefrontal cortex (Prasser et al., 2014). Common clinical paradigms include left-sided high-frequency stimulation to enhance left DLPFC excitability, right-sided low-frequency stimulation to suppress putative right-hemisphere hyperactivity, sequential bilateral “left-high/right-low” combinations, and patterned burst protocols such as intermittent theta-burst stimulation (iTBS) intended to induce rapid long-term potentiation (LTP)-like plasticity (Prasser et al., 2014; Huang et al., 2005; Blumberger et al., 2018; Suppa et al., 2016). Randomized trials and network meta-analyses indicate that multiple active rTMS modalities are superior to sham for acute symptom reduction, and that iTBS can achieve clinical outcomes comparable to conventional high-frequency stimulation while drastically shortening treatment time (Buhle et al., 2013). However, direct comparative evidence across hemispherically distinct stimulation strategies remains limited and partially inconsistent, with some studies reporting comparable short-term efficacy across protocols, while others suggest differential response patterns depending on stimulation laterality, frequency, or patient characteristics (Lang et al., 2011; Blumberger et al., 2018). As a result, the short-term clinical effectiveness of these commonly used protocols−and the sources of their heterogeneous response profiles−remains incompletely resolved. This uncertainty highlights a central knowledge gap in the field: how different hemispherically informed rTMS/iTBS protocols compare in their short-term antidepressant efficacy.

In parallel with efforts to optimize stimulation protocols, increasing attention has been directed toward identifying cognitive mechanisms that might account for variability in clinical response. Cognitive reappraisal-the intentional reinterpretation of affective stimuli to lessen their emotional impact-is a canonical prefrontal-mediated emotion regulation strategy and a compelling candidate mediator of rTMS efficacy in MDD (Kluetsch et al., 2012). Behaviorally, individuals with MDD use reappraisal less frequently and less successfully than non-depressed controls, and poorer reappraisal capacity correlates with symptom severity and impaired functional outcomes (Ge et al., 2022; Li et al., 2023; Neacsiu et al., 2021). Neuroimaging meta-analyses of reappraisal consistently implicate left lateral prefrontal regions (VLPFC and DLPFC) in the implementation and selection of reinterpretation strategies, with effective reappraisal associated with down-regulation of amygdala responses via prefrontal control signals (Blumberger et al., 2022). Importantly, recent neuromodulation studies demonstrate that directly stimulating lateral prefrontal nodes modifies the neural substrates of reappraisal: TMS-fMRI and TMS-EEG experiments show that left-prefrontal stimulation can alter downstream amygdala reactivity and enhance electrophysiological markers associated with cognitive control during reappraisal tasks (Philip et al., 2017; Paneva et al., 2025; Plewnia et al., 2021). However, existing evidence remains fragmented: many studies focus on reappraisal-related neural effects without assessing clinical outcomes, whereas clinical trials rarely examine whether short-term symptom improvement is accompanied by changes in emotion-regulation capacity. Thus, whether modulation of cognitive reappraisal contributes to short-term antidepressant response−and whether such modulation differs across hemispherically informed stimulation protocols−remains an open mechanistic question rather than an established causal pathway.

On this basis, the present comparison is not intended to establish the superiority of bilateral over unilateral stimulation, but to dissociate their short-term clinical effects and cognitive mechanisms, given that both approaches remain widely used in routine practice. We tested the hypothesis that stimulation mode−intermittent theta-burst stimulation (iTBS) targeted to left lateral prefrontal cortex versus a sequential bilateral “left-high/right-low” rTMS regimen−will differentially engage the prefrontal mechanisms that subserve cognitive reappraisal and therefore produce distinct profiles of change in both reappraisal ability and depressive symptoms. Mechanistically, iTBS is designed to induce rapid, focal LTP-like plasticity in the stimulated cortex and its functionally connected network, thereby preferentially potentiating left-hemisphere control signals that implement reinterpretation strategies (Chen et al., 2021; Lu et al., 2023; Murgaš et al., 2023); by contrast, a sequential left-high/right-low protocol may exert therapeutic effects via a mixture of left-hemisphere facilitation and right-hemisphere down-regulation that restores interhemispheric balance but with a different spatial and temporal signature of plasticity (Prasser et al., 2014). We therefore prespecified the change in depression severity as the primary outcome, with clinical (cognitive reappraisal score) secondary endpoints to test whether protocol-specific modulation of left-prefrontal control predicts symptomatic improvement. If iTBS produces greater and faster gains in reappraisal capacity than sequential bilateral stimulation, and if those gains mediate symptom reduction, the result would furnish a concrete, mechanistic rationale for selecting stimulation paradigms based on the dominant cognitive-affective deficit of the patient−a step toward phenotype-matched, mechanism-driven neuromodulation for MDD (Hühne et al., 2023).

## 2 Materials and methods

### 2.1 Study subjects

**FIGURE 1:**
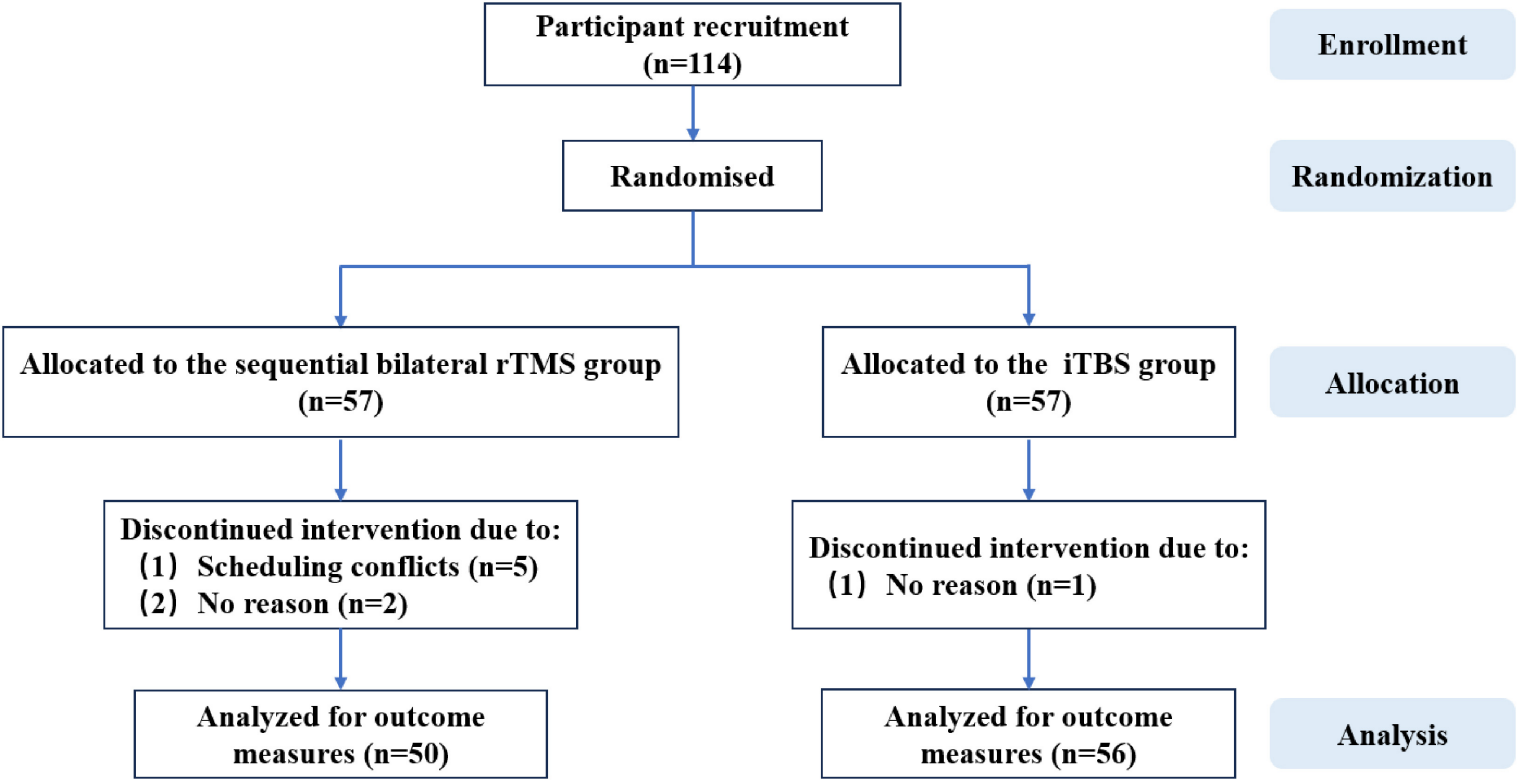
CONSORT flow diagram.

The 114 subjects with MDD in this study were recruited from February 2024 to June 2025 in Chengdu, China. The study was approved by the ethics committee of Chengdu 4th People’s Hospital, and the clinical trial registration ID is NCT06417437. All participants provided written informed consent before any procedures were per-formed. Figure 1 illustrates the flow diagram of the present study.

The inclusion criteria for the depressive disorder participants were as follows (Pu et al., 2025): (1) meet the DSM-V diagnostic criteria for “Depressive Disorders” (Tolentino and Schmidt, 2018); (2) Han ethnicity, age ≥ 18 years; (3) junior high school education level or above; (4) right-handed; (5) HAMD-17 score ≥ 17; (6) medication use: no psychiatric-related medications use in the past 3 months or a stable selective serotonin reuptake inhibitor (SSRI) regimen for at least 3 months prior to and throughout the physical regulation intervention; and (7) willing to participate in the experiment, accept treatment, and sign an informed consent form. None of the participants met criteria for treatment-resistant depression. Participants were either antidepressant-naïve or had received no more than one prior adequate antidepressant trial.

The exclusion criteria for all individuals were as follows (Pu et al., 2025): (1) combined or previous history of organic brain diseases or severe brain trauma, personal or family history of epilepsy; (2) severe cardiac, hepatic, or renal dysfunction; (3) patients with severe physical diseases; (4) history of substance dependence or abuse (alcohol, cocaine, drugs, etc.); (5) concurrent other psychiatric disorders; (6) pregnancy or lactation; (7) use of electroconvulsive therapy (ECT) without seizure, transcranial magnetic stimulation (TMS), or other physical treatments within the past 6 months; (8) implanted auto-nomic nerve stimulators; (9) implanted electronic or metal devices (such as pacemakers, defibrillators, stents, orthopedic steel plates, etc.), or those who have undergone ven-triculoperitoneal shunting; and (10) significant visual or auditory impairments that prevent completion of neuropsychological testing and scale assessments.

### 2.2 Sample size calculation

Sample size was calculated based on the primary outcome, defined as the change in HAMD score from baseline to the end of the 2-weeks treatment period. Based on data from a pilot study, the mean change in HAMD score was 8.27 in the sequential bilateral rTMS group and 13.02 in the iTBS group, with a standard deviation of 6.81. To achieve 90% statistical power at a two-sided significance level of α = 0.05, a minimum of 45 participants per group was required. Assuming an anticipated dropout rate of 20%, the target sample size was increased to 57 participants per group, resulting in a total planned sample of 114 participants. Sample size calculations were performed using G*Power version 3.1.9.7.

### 2.3 Clinical and psychological evaluation

Demographic data were collected at baseline, and clinical and psychological assessments were conducted both at baseline and at the end of the 2-weeks treatment. Two senior psychology experts, each with >10 years of work experience, independently performed the psychological evaluation without knowledge of the participants’ clinical diagnosis, then another senior psychiatrist reviewed the assessment results. The following scales were used, including the following (Pu et al., 2025): the Pittsburgh Sleep Quality Index (PSQI) (Chang et al., 2021), the trail making test (TMT) (Zhao et al., 2013), the digit span test (DST) (Leung et al., 2011), the Hamilton anxiety rating scale (HAMA) (Maier et al., 1988), the Hamilton depression rating scale (HAMD) (Bech et al., 2014), the Social Support Rating Scale (SSRS) (Kong et al., 2024), the Beck scale for suicide ideation (BSSI) (Strumila et al., 2024), the Snaith–Hamilton Pleasure Scale (SHAPS) (Wong et al., 2024), the Emotion regulation questionnaire (ERQ) (Wong et al., 2024), a Self-Rating Anxiety Scale (SAS) (Dunstan and Scott, 2020), a self-rating depression scale (SDS) (Zung, 1965). The ERQ was used to assess emotion regulation and comprises two subscales: the Cognitive Reappraisal subscale (6 items) and the Expressive Suppression subscale (4 items), both rated on a 7-point Likert scale, with higher scores indicating greater use of the respective strategy.

### 2.4 Randomization and blinding

Participants were randomly assigned in a 1:1 ratio to either the sequential bilateral rTMS group (*n* = 57) or the iTBS group (*n* = 57) using a computer-generated simple randomization sequence. Allocation concealment was ensured by sealed, opaque, and sequentially numbered envelopes prepared by an independent researcher who was not involved in treatment delivery or outcome assessment.

Outcome assessors were blinded to treatment assignment. Prior to the start of the study, they were explicitly instructed not to discuss participants’ diagnostic information either with the participants themselves or with any individuals who were aware of the group assignments. Participants were informed that they would receive one of two active TMS protocols but were unaware of which specific stimulation protocol they were assigned to. Due to the inherent differences in stimulation schedules and parameters between the two protocols, treating therapists were not blinded to treatment allocation.

### 2.5 TMS intervention protocol

In this study, transcranial magnetic stimulation (TMS) was delivered using the YRD CCY-1 stimulator (Wuhan Yiruide Co., Wuhan, China). All participants underwent continuous treatment for two consecutive weeks. Target localization for all stimulation protocols was performed using a scalp-based method guided by the international 10–20 EEG system, with the left dorsolateral prefrontal cortex (DLPFC) corresponding approximately to F3 and the right DLPFC corresponding approximately to F4. The resting motor threshold (RMT) for each participant was determined according to standard procedures prior to stimulation. The sequential bilateral rTMS protocol was administered once daily, whereas the left-sided iTBS protocol was delivered twice daily, reflecting their respective stimulation schedules.

#### 2.5.1 Sequential bilateral rTMS group

The stimulation sites were the left and right dorsolateral prefrontal cortices (L-DLPFC and R-DLPFC). For the sequential bilateral rTMS protocol, stimulation was delivered in a fixed order within each session, with intermittent theta-burst stimulation (iTBS) applied first to the L-DLPFC, followed immediately by low-frequency rTMS applied to the R-DLPFC. For the L-DLPFC, iTBS was applied using a figure-eight coil positioned tangentially to the scalp, with the coil oriented at approximately 45° relative to the midline, in accordance with standard clinical recommendations for left DLPFC stimulation, at 90% of the resting motor threshold (RMT). Each burst consisted of three pulses at 50 Hz, repeated every 200 ms (5 Hz), forming a 2 s train followed by an 8 s inter-train interval. This sequence was repeated 20 times, yielding a total of 600 pulses per session, with a total duration of 3 min 20 s. For the R-DLPFC, low-frequency rTMS was delivered at 1 Hz using the same figure-eight coil configuration, at 100% of RMT. Each session consisted of 60 trains of 20 pulses each, with 1 s inter-train intervals, totaling 1200 pulses and lasting approximately 21 min. Both stimulations were administered once daily, 5 days per week (Monday to Friday) for two consecutive weeks, for a total of 10 treatment sessions.

#### 2.5.2 iTBS group

The stimulation site was the left dorsolateral prefrontal cortex (L-DLPFC). Intermittent theta-burst stimulation (iTBS) was applied using a figure-eight coil positioned parallel to the skull and in close contact with the scalp, at 90% of the resting motor threshold (RMT). The stimulation parameters consisted of bursts of three pulses at 50 Hz, repeated every 200 ms (5 Hz theta frequency) to form a 2 s train followed by an 8 s rest. This sequence was repeated 60 times, resulting in a total of 1800 pulses per session, with a total stimulation duration of approximately 10 min. Each participant received two iTBS sessions per day, separated by an interval of 50 min, 5 days per week (Monday to Friday) for two consecutive weeks, totaling 20 treatment sessions. All sessions were conducted by trained technicians under physician supervision. Coil positioning was guided by the international 10–20 EEG system, with the left DLPFC corresponding approximately to F3. Participants were seated comfortably and monitored throughout each session to ensure safety and tolerability.

### 2.6 Outcome measures

The primary outcome was the change in HAMD score from baseline to the end of the 2-weeks treatment period. Secondary outcomes included changes from baseline to post-treatment in cognitive reappraisal score, HAMA, SHAPS, BSSI, PSQI, TMT, DST, SSRS, SAS, and SDS. All change scores were calculated as baseline minus post-treatment values.

### 2.7 Safety measures

Adverse effects were systematically monitored and recorded throughout the intervention period. At each stimulation session, participants were actively queried by study physicians regarding the occurrence of any discomfort or unexpected symptoms, and spontaneous reports from participants were also documented. Expected TMS-related adverse events, including headache, scalp discomfort, dizziness, and fatigue, were specifically assessed at each visit. Serious adverse events (SAEs) and instances of treatment discontinuation were documented and evaluated by the study physician. All reported adverse events were classified according to severity and their relationship to the intervention.

### 2.8 Statistical analysis

Statistical analyses were performed using SPSS version 21.0 (SPSS, Chicago, USA). Continuous variables were expressed as mean ± standard deviation (SD) if normally distributed, and as median (interquartile range, IQR) if not normally distributed. Categorical variables were expressed as counts and percentages. Normality and homogeneity of variance were assessed for all continuous variables prior to group comparisons. Group comparisons were performed using independent-sample *t*-tests for continuous variables that satisfied parametric assumptions; otherwise, the Mann–Whitney U test was applied. Categorical variables were compared using the Chi-square test. Correlation analyses were conducted using Pearson correlation to examine linear relationships between continuous variables. In addition, generalized linear models (GLMs) were used to assess the associations between HAMD and cognitive reappraisal score more robustly. Statistical significance was set at *P* < 0.05. Bonferroni correction was applied for multiple comparisons in secondary outcomes, with a significance threshold set at *P* < 0.005 (0.05/11).

## 3 Results

### 3.1 Characteristics of participants

A total of 114 participants were enrolled in the study, of whom 106 completed the trial. At baseline, the two treatment groups–sequential bilateral rTMS and iTBS–were well matched with respect to demographic characteristics and psychobehavioral scale scores. There were no significant differences between groups in terms of age, sex, disease duration, or baseline assessments of depression, anxiety, sleep quality, social support, anhedonia, suicidal ideation, and emotion regulation (all *P* > 0.05) (Table 1 and Supplementary material).

**TABLE 1:** Comparison of baseline characteristics between iTBS and sequential bilateral rTMS groups at baseline.

Baseline characteristics	MDD group (*n* = 106)	MDD group		*P*-value
		Sequential bilateral rTMS group (*n* = 50)	iTBS group (*n* = 56)	
Gender (Female, %)	72 (67.9)	34 (68)	38 (67.86)	1.00
Age (years)	31.56 ± 9.44	31.42 ± 9.19	31.68 ± 9.74	0.889
Height (cm)	163.13 ± 7.65	163.88 ± 8.10	162.43 ± 7.22	0.335
Weight (kg)	58.24 ± 12.31	58.73 ± 11.98	57.778 ± 12.70	0.696
Disease duration (years)	4.47 ± 0.92	4.43 ± 0.96	4.51 ± 0.89	0.439
Self-rating depression scale	69.81 ± 8.47	68.98 ± 9.78	70.55 ± 7.10	0.342
Self-Rating Anxiety Scale	60.71 ± 10.83	58.92 ± 11.78	62.30 ± 9.73	0.114
Cognitive reappraisal score	18.56 ± 5.17	17.94 ± 4.29	19.11 ± 5.83	0.248
Expressive suppression score	18.23 ± 5.71	17.24 ± 5.72	19.11 ± 5.6	0.093
Snaith–Hamilton Pleasure Scale	33.20 ± 6.14	33.28 ± 7.24	33.13 ± 5.0	0.897
Pittsburgh Sleep Quality Index	12.74 ± 3.91	12.40 ± 4.25	13.04 ± 3.59	0.406
Beck scale for suicide ideation in the past week	7.07 ± 7.60	7.82 ± 8.46	6.39 ± 6.75	0.337
Social Support Rating Scale	30.80 ± 7.70	30.06 ± 7.23	31.46 ± 8.10	0.351
Hamilton depression rating scale	23.27 ± 3.69	22.62 ± 3.73	23.86 ± 3.59	0.085
Hamilton anxiety rating scale	17.04 ± 5.28	16.16 ± 5.34	17.82 ± 5.15	0.106
Digit span forward score	7.55 ± 1.52	7.54 ± 1.45	7.55 ± 1.60	0.964
Digit span backward score	4.55 ± 1.81	4.50 ± 1.68	4.59 ± 1.92	0.801
Trail making test part A	32.91 ± 22.30	35.85 ± 21.78	30.29 ± 22.62	0.201
Trail making test part B	89.99 ± 38.14	89.63 ± 35.62	90.32 ± 40.57	0.928

*P*-value represents the statistical significance of differences in baseline characteristics between the sequential bilateral rTMS group and the iTBS group.

### 3.2 Efficacy outcomes

#### 3.2.1 Primary outcome

**FIGURE 2:**
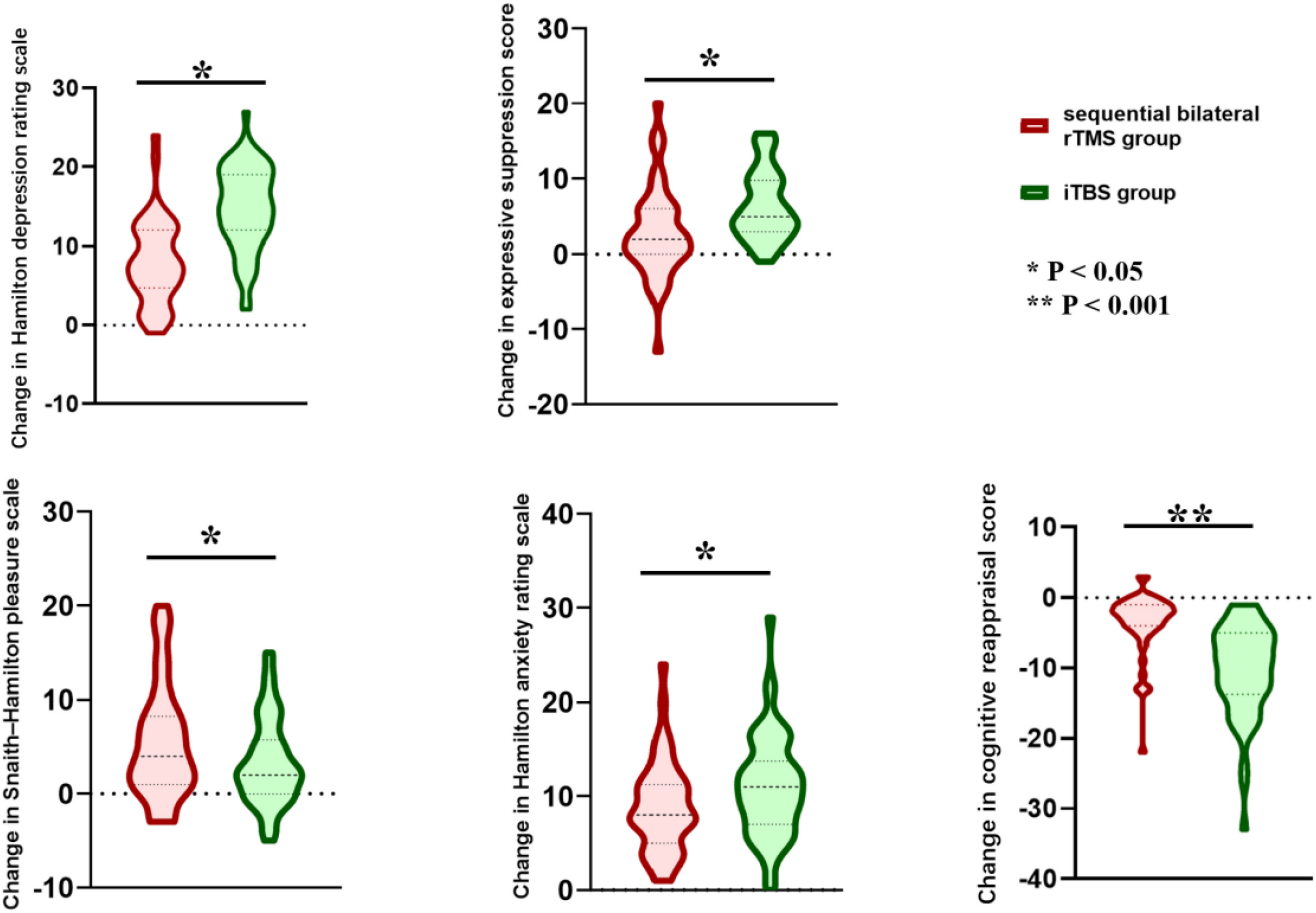
Comparison of differences before and after treatment. * indicates *p* < 0.05 and ** indicates *p* < 0.001.

Compared with the sequential bilateral rTMS group, participants receiving iTBS exhibited significantly greater improvements across multiple clinical and psychological domains following the 2-weeks intervention. The reduction in depressive symptom severity, as indexed by the HAMD score, was significantly larger in the iTBS group than in the sequential bilateral rTMS group (14.88 ± 5.25 vs. 7.88 ± 5.26; *P* < 0.001) (Figure 2).

#### 3.2.2 Secondary outcomes

Compared with the sequential bilateral rTMS group, improvement in cognitive reappraisal ability was more pronounced in the iTBS group (−10.36 ± 6.49 vs. −3.54 ± 4.41; *P* < 0.001, significant after Bonferroni correction). For expressive suppression, the iTBS group showed a greater reduction than the rTMS group (6.39 ± 4.54 vs. 2.88 ± 5.93; *P* = 0.001, significant after Bonferroni correction). The iTBS group demonstrated a more substantial decrease in anxiety symptoms assessed by HAMA (10.96 ± 5.34 vs. 8.86 ± 4.78; *P* = 0.036). These findings indicate that iTBS may induce broader and more rapid clinical and emotional benefits than sequential bilateral rTMS in patients with major depressive disorder. In addition, the sequential bilateral rTMS group demonstrated a greater alleviation of anhedonia as reflected by lower SHAPS scores (5.44 ± 6.02 vs. 3.29 ± 4.54; *P* = 0.039) (Figure 2). However, changes in HAMA and SHAPS scores did not remain significant after Bonferroni correction.

### 3.3 Interrelationships among depression scale and psychological measures in patients with MDD

#### 3.3.1 Correlations of self-rating depression scale with other measures

**FIGURE 3:**
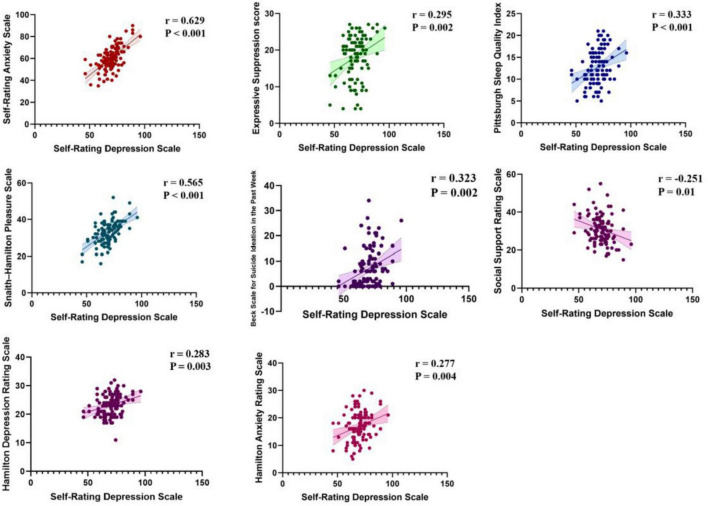
Correlations of self-rating depression scale with other measures.

The SDS were examined for their associations with various clinical and psychological measures. For anxiety - related scales, a strong positive correlation was observed between SDS and the Self–Rating Anxiety Scale (*r* = 0.629, *P* < 0.001). In terms of emotional and cognitive regulation, SDS was positive correlated with the expressive suppression score (*r* = 0.295, *P* = 0.002). Regarding hedonic capacity, a positive correlation existed between SDS and the Snaith–Hamilton Pleasure Scale (*r* = 0.565, *P* < 0.001). For sleep quality, SDS was positively correlated with the Pittsburgh Sleep Quality Index (*r* = 0.333, *P* < 0.001), as SDS was negatively correlated with the Social Support Rating Scale (*r* = −0.251, *P* = 0.01). When looking at other depression assessment scales, SDS was positively correlated with both the Hamilton depression rating Scale (*r* = 0.283, *P* = 0.003) and the days scale for suicide ideation in the last week (*r* = 0.323, *P* = 0.002). Additionally, SDS showed a positive correlation with the HAMA scale (*r* = 0.277, *P* = 0.004) (Figure 3). No correlation was found between SDS and the cognitive reappraisal score (*r* = −0.153, *P* = 0.117).

#### 3.3.2 Correlations of Hamilton depression rating scale with other measures

**FIGURE 4:**
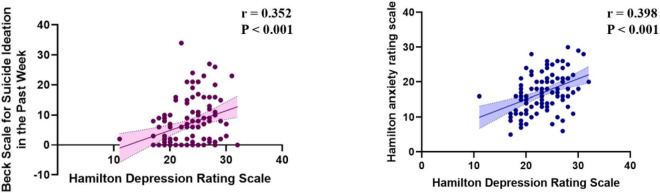
Correlations of Hamilton depression rating scale with other measures.

The HAMD scores were examined for their associations with various clinical and psychological measures. In terms of suicide - related measures, a positive correlation existed between HAMD and the Beck Scale for Suicide Ideation in the Past Week (*r* = 0.352, *P* < 0.001). For anxiety - related scales, a positive correlation was observed between HAMD and the HAMA scale (*r* = 0.398, *P* < 0.001) (Figure 4).

### 3.4 Correlation analysis between post-treatment HAMD and cognitive reappraisal score changes

A generalized linear model was used to examine the relationship between changes in HAMD scores and changes in cognitive reappraisal scores, with HAMD change as the dependent variable and cognitive reappraisal change as the independent variable. In the overall sample, there was a significant negative association between the two measures (*B* = −0.447, *P* < 0.001). In subgroup analyses, no significant relationship was observed in the sequential bilateral rTMS group (*B* = 0.002, *P* = 0.991), whereas a significant negative association was found in the iTBS group (*B* = −0.324, *P* = 0.001), indicating that greater improvements in cognitive reappraisal were associated with larger reductions in depressive symptoms in the iTBS-treated participants.

### 3.5 Safety

No SAEs were reported in either treatment group during the study period. In the sequential bilateral rTMS group, mild and transient adverse events were reported, including headache (*n* = 3) and dizziness (*n* = 1). In the iTBS group, headache was reported by two participants (*n* = 2), and no other adverse events were observed. All reported adverse events were self-limited, did not require medical intervention, and did not lead to treatment discontinuation.

## 4 Discussion

In this randomized controlled trial, we compared the efficacy of sequential bilateral rTMS and iTBS in patients with MDD. Our findings demonstrate that iTBS elicited more pronounced improvements across multiple domains, including depressive symptom severity, cognitive reappraisal ability, expressive suppression, and anxiety, compared with sequential bilateral rTMS over the 2-weeks intervention period. Importantly, changes in cognitive reappraisal were significantly associated with reductions in depressive symptoms in the iTBS group; however, these associations are correlational and do not imply causal mediation. Nonetheless, both interventions produced clinically meaningful improvements, highlighting that sequential bilateral rTMS also confers therapeutic benefit, albeit with a potentially different temporal pattern of clinical response.

Baseline demographic and clinical characteristics, including age, sex, depression severity, anxiety, sleep quality, social support, anhedonia, suicidal ideation, and emotion regulation scores, were comparable between groups, minimizing potential confounding. These baseline associations underscore the importance of targeting both affective and cognitive-emotional regulation processes when evaluating treatment response.

In the present study, iTBS demonstrated superior efficacy in alleviating depressive symptoms compared with sequential bilateral rTMS. This finding is consistent with prior trials showing that standard iTBS targeting the L-DLPFC produces antidepressant effects comparable to, or exceeding, those of conventional high-frequency rTMS, while offering greater treatment efficiency (Blumberger et al., 2018). Moreover, studies employing accelerated iTBS protocols have demonstrated that increased session density can lead to rapid and robust symptom improvement, highlighting the potential of iTBS to induce early therapeutic effects (Cole et al., 2020). In contrast, classical bilateral rTMS protocols−typically combining high-frequency stimulation of the L-DLPFC with low-frequency stimulation of the R-DLPFC−have demonstrated antidepressant efficacy comparable to unilateral stimulation (Fitzgerald et al., 2006), but have not consistently shown superiority in terms of clinical outcomes or speed of response, and typically require longer session durations per day (Chen et al., 2014). Notably, Prasser et al. (2014) reported a trend toward greater symptom improvement with bilateral theta-burst stimulation compared with bilateral rTMS at follow-up, suggesting that bilateral TBS-based approaches may represent a promising alternative to conventional bilateral rTMS protocols. Notably, although overall symptom reduction was greater in the iTBS group, the relatively greater improvement in anhedonia observed in the sequential bilateral stimulation group may reflect differential engagement of reward-related neural circuits. Anhedonia has been closely linked to dysfunction within frontostriatal networks, and bilateral prefrontal modulation may exert a more balanced influence on these circuits than unilateral stimulation alone. This interpretation is consistent with prior evidence that prefrontal stimulation can indirectly modulate striatal and limbic activity via network-level mechanisms (Russo and Nestler, 2013; Fox et al., 2012). Taken together, these findings, in conjunction with the present results, support the notion that iTBS-based interventions−particularly those targeting the L-DLPFC−may offer a more efficient and clinically advantageous neuromodulation strategy for MDD, while underscoring the need for future trials directly comparing dose-matched and protocol-matched bilateral and unilateral TBS paradigms.

The observed differences in emotional and cognitive outcomes between the two modalities provide insight into protocol-specific mechanisms of action. iTBS may rapidly prime the left prefrontal cortex to support the acquisition and implementation of adaptive reappraisal strategies, translating to faster and more robust clinical improvement (Cristancho et al., 2020; Liston et al., 2014; Hayatbini et al., 2021). Sequential bilateral rTMS, while slower in onset, may promote global network reorganization and interhemispheric balance, offering a complementary route to symptom amelioration. Taken together, these findings highlight that the choice of stimulation protocol can be informed by the dominant cognitive-affective deficits of individual patients, aligning with the paradigm of mechanism-driven, phenotype-matched neuromodulation for MDD (Fitzgerald et al., 2016).

The present findings have several important clinical implications. Although the twice-daily intervention protocol may pose logistical challenges in routine clinical practice, such an approach is likely feasible in structured rehabilitation settings or short-term, supervised intervention programs, particularly among individuals at early disease stages. From a cost perspective, the non-invasive nature of the intervention may reduce medical risk and monitoring burden compared with pharmacological strategies; however, cumulative time and personnel requirements may increase overall costs, underscoring the need for future cost-effectiveness evaluations and optimized delivery models. Patient adherence may be facilitated by the favorable safety profile and potential perceived cognitive benefits, yet treatment burden remains a relevant consideration for long-term implementation. Importantly, our results suggest that patient selection strategies informed by specific cognitive or regulatory deficits−such as impaired reappraisal-related processes−may enhance both clinical efficacy and resource efficiency. This aligns with emerging precision-oriented approaches in cognitive intervention and may improve the translational potential of the present findings.

Several limitations should be acknowledged. First, the absence of a sham control condition precludes disentangling stimulation-specific effects from nonspecific placebo or expectancy effects; therefore, treatment effects should be interpreted with caution. Second, the intervention period was limited to 2 weeks, preventing assessment of the long-term durability of treatment effects. Third, although changes in cognitive reappraisal were associated with symptom improvement, causal mediation cannot be conclusively inferred from the current design, and relevant covariates (e.g., baseline HAMD score, baseline reappraisal score, medication status, age, and sex) may have influenced these associations. Fourth, the iTBS and sequential bilateral rTMS protocols differed in total stimulation dose, daily session frequency, and stimulation pattern, which limits isolation of dose-response effects and may partly account for between-group differences. Finally, the modest sample size, incomplete blinding, single-center design (including restriction to Han ethnicity and a relatively young sample), and short follow-up constrain statistical power and generalizability. Future sham-controlled, dose-matched studies with larger samples, longer follow-up, and mediation or mixed-effects modeling are warranted.

In conclusion, our results indicate that iTBS targeting the left DLPFC produces more pronounced improvements in depressive symptoms and cognitive reappraisal compared with sequential bilateral rTMS over the 2-weeks intervention period, whereas both modalities confer meaningful clinical benefits. Observed changes in cognitive reappraisal were associated with reductions in depressive symptoms, suggesting that improvements in emotion regulation capacity may accompany clinical symptom relief. Future studies incorporating neurophysiological or neuroimaging measures are warranted to directly investigate the neural substrates underlying these clinical and cognitive changes and to inform individualized neuromodulation strategies in MDD.

## Data Availability

The raw data supporting the conclusions of this article will be made available by the authors, without undue reservation.
