# Supplementary material for: Comparative effects of intermittent theta-burst stimulation and sequential bilateral rTMS on depression and emotion regulation in major depressive disorder: a randomized controlled trial
Source: Front Neurosci. 2026 Feb 13;20:1756383. doi: 10.3389/fnins.2026.1756383 (PMC12946152; doi:10.3389/fnins.2026.1756383)
Supplement: Supplementary file 1 [file Table_1.docx]

Supplementary Material

# Supplementary Materials and Methods

**Existing medications of participants**

During the intervention period, 36 participants in the sequential bilateral rTMS group and 41 participants in the iTBS group were receiving a stable dose of escitalopram (10-15 mg/day). In contrast, 28% of participants in the sequential bilateral rTMS group and 27% in the iTBS group had not received any psychiatric-related medications in the three months preceding the study. There was no significant difference in medication status between the two groups (*P* = 0.889).

**Table S1.** Comparison of medication status between iTBS and sequential bilateral rTMS groups.

|  | **sequential bilateral rTMS group**  **(n = 50)** | **iTBS group**  **(n = 56)** | ***P* value** |
| --- | --- | --- | --- |
| No psychiatric-related medications use, n (%) | 14 (28) | 15 (27) | 0.889 |
| Escitalopram use, n (%) | 36 (72) | 41 (73) |  |

*Note:* *rTMS: repetitive transcranial magnetic stimulation; iTBS: intermittent theta-burst stimulation.*
